# The Utility of Intravenous Acetaminophen in the Perioperative Period

**DOI:** 10.3389/fpubh.2013.00025

**Published:** 2013-08-06

**Authors:** Jason B. O’Neal

**Affiliations:** ^1^Department of Anesthesia, Critical Care, and Pain Medicine, Beth Israel Deaconess Medical Center, Harvard Medical School, Boston, MA, USA

**Keywords:** acetaminophen, intravenous, postoperative, surgery

## Abstract

Intravenous acetaminophen (IVA) has rapid and effective analgesic properties. Recent studies have shown several benefits of using IVA perioperatively. However, due to its relatively high cost and limited clinical data concerning its efficacy compared with other agents, physicians are hesitant to use IVA in the perioperative period. This brief review examines the utility of this medication in the perioperative period and highlights future areas of clinical and epidemiological research regarding its use.

## Introduction

Intravenous acetaminophen (IVA), or paracetamol, was made available in Europe in 2002, but only approved for use in the United States in November of 2010. IVA has both rapid and effective analgesic properties and is considered to be safe and well tolerated (1). Physicians are sometimes hesitant to use IVA in the perioperative period due to its cost and also the relatively limited amount of available data and supporting studies regarding its efficacy. This brief review examines several recent studies of IVA and explores the utility of this medication in the perioperative period.

## Mechanism of Action

The exact mechanism by which acetaminophen produces analgesia has yet to be fully elucidated (2). Suggested mechanisms include inhibition of the synthesis of cyclooxygenases which are involved in prostaglandin production. It also may act on serotonin pathways that regulate spinal nociception. Following surgery, patients release inflammatory mediators such as histamine, leukotrienes, cytokines, and prostaglandins. Afferent neurons release glutamate, substance P, neurokinins, and other peptides (2). IVA may inhibit the formation of these inflammatory markers and block pain pathways to achieve an analgesic effect and reduce pain in patients postoperatively.

## Postoperative Pain

Postsurgical pain has been reported in over 70% of patients, with 31% of patients reporting severe pain and 47% experiencing moderate levels (2, 3). Additionally, inadequate pain control contributes to several complications after surgery including a higher incidence of myocardial infarction, impaired wound healing, and poor respiratory effort possibly contributing to postoperative pneumonia (3). However, alleviating pain with current analgesics such as opiates is not without adverse effects. Opioids may cause drowsiness, postoperative nausea and vomiting, ileus, respiratory depression, and bladder dysfunction (4). The addition of IVA was shown in several studies to decrease the amount of opiates consumed after surgery, but the reduction of adverse effects associated with opioids has mixed results (2, 4, 5).

## Intravenous Versus Oral Acetaminophen

Comparatively, the cost of IVA with oral acetaminophen is several folds higher, which inhibits many physicians from using the intravenous agent. Also, insurance companies reimburse for a fraction of the cost, resulting in a loss for hospitals. Although cost may be an issue, there is evidence which favors the intravenous formulation over the oral agent. The bioavailability of IVA in cerebrospinal fluid compared with oral acetaminophen after the administration of 1 g over 6 h is 24.9 versus 14.2 μg · h/mL (6). The intravenous route also produces a higher plasma concentration than oral administration and peaks much faster after only 15 min compared with >45 min with oral acetaminophen (3). Early plasma concentrations of acetaminophen are highly variable when administered orally and may remain in the subtherapeutic range much longer than IVA (3). Currently, the correlation of adequate pain control with cerebrospinal or plasma levels of intravenous and oral agents has yet to be investigated. Intravenous administration also produces less of acetaminophen’s toxic metabolite (*N*-acetyl-*p*-benzoquinoneimine) than when ingested orally, making IVA a safer alternative. Intravenous infusion produces a peak acetaminophen concentration in the liver estimated to be 50% less than the same oral dose (7). Nonetheless the potential for overdose still exists with IVA and must be carefully monitored in high-risk patients, such as children and those with liver disease.

## Intravenous Acetaminophen and Postsurgical Analgesia

Studies have evaluated the use of IVA in surgical patients undergoing cesarean sections, hysterectomies, coronary artery bypass grafting, hip or knee replacements, thyroidectomies, and other surgeries (8). A study looking at IVA use (1 g every 6 h) showed a decrease in morphine consumption by 46% (*p* = 0.0003) on postoperative day one after total hip replacement or total knee replacement and found an increased time to receive rescue morphine (hip replacement, 3.9 h and knee replacement, 2.1 h) compared with placebo (0.8 h) (5). Additionally, a recent review of 14 randomized controlled trials found that patients receiving IVA had improved analgesia in 12 of the studies examined (8). Other studies in hysterectomy and laminectomy patients suggest that patients receiving IVA may have a reduction in postoperative nausea and vomiting, although this has yet to be formally evaluated (8). Furthermore, preemptive (before surgical incision) versus preventive (after surgical incision) use of IVA was not shown to have a difference in pain control, but both groups had a decrease in pain scores 6 h after surgery compared with placebo (*p* < 0.001) (9).

## Intravenous Acetaminophen in the Intensive Care Unit

The use of IVA in the ICU setting also has been investigated. A study including patients admitted to the ICU after major abdominal or pelvic surgery examined the amount of opioids used, time to extubation, and opioid-related adverse effects in patients receiving 1 g of IVA every 6 h over 24 h (4). Time to extubation was reduced from an average of 204.5 ± 112.7 min in the referent group compared with only 64.3 ± 40.6 min in the IVA group (*p* < 0.01). In addition, total opiate consumption was significantly reduced in the IVA group compared with referents. Other studies based in the ICU have focused on delirium in cardiac surgery patients. Reports have estimated that over 50% of patients undergoing major cardiac surgery experience delirium during admission and postoperative pain and excessive opioid consumption potentially lead to this complication (10). Although further research is necessary, the use of IVA in this patient population may reduce the amount of opiate consumption and subsequently lead to a reduction in the incidence of delirium.

## Conclusion

The use of IVA is approved by the Food and Drug Administration for the management of mild to moderate pain, the management of moderate to severe pain with opioid analgesic adjuncts, and fever reduction in adults and children over the age of two (11). Recent studies have shown several benefits of using IVA perioperatively, including a reduction in opiate consumption, improved analgesia, and reduced postoperative nausea and vomiting. Studies have not shown a decrease in the incidence of opioid-related adverse effects, although this may be due to inadequate sample sizes and difficulty in recording adverse events. Also, the cost effectiveness of using IVA has not been extensively evaluated in surgical patients. It is known that the cost of IVA is much higher than oral acetaminophen and many hospitals do not support its use during the perioperative period for this reason. Studies comparing IVA to oral acetaminophen are lacking and have not shown a benefit in using IVA compared with the oral formulation (12). More research is needed in this area to better assess the utility of IVA and to further evaluate the potential reduction in opioid adverse effects. Additionally, future clinical and epidemiologic research should examine the efficacy of IVA compared with oral acetaminophen to determine the best modality to control postoperative pain and reduce complications. Since cost is one of the limiting factors for giving patients IVA, comparative effectiveness studies comparing IVA to oral acetaminophen which focus on time to post-anesthesia care unit discharge in both inpatient and outpatient settings are warranted. IVA has the potential to be a very versatile and worthwhile addition to achieving adequate postoperative analgesia in patients undergoing a wide variety of surgical procedures and in numerous hospital settings.

## Conflict of Interest Statement

The authors declare that the research was conducted in the absence of any commercial or financial relationships that could be construed as a potential conflict of interest.
